# The frequency of re-planning and its variability dependent on the modification of the re-planning criteria and IGRT correction strategy in head and neck IMRT

**DOI:** 10.1186/1748-717X-9-175

**Published:** 2014-08-11

**Authors:** Markus Stoll, Kristina Giske, Jürgen Debus, Rolf Bendl, Eva Maria Stoiber

**Affiliations:** Department of Medical Physics in Radiation Oncology, DKFZ, INF 280, 69120 Heidelberg, Germany; Department of Radiation Oncology, University Hospital, Heidelberg, Germany; Faculty of Medical Informatics, Heilbronn University, Heilbronn, Germany

**Keywords:** Image-guided radiation therapy, Head and neck cancer, Deformable image registration, Dose variations, Re-planning criteria, Re-planning frequency

## Abstract

**Background:**

To analyse the frequency of re-planning and its variability dependent on the IGRT correction strategy and on the modification of the dosimetric criteria for re-planning for the spinal cord in head and neck IG-IMRT.

**Methods:**

Daily kV-control-CTs of six head and neck patients (=175 CTs) were analysed. All volumes of interest were re-contoured using deformable image registration. Three IGRT correction strategies were simulated and the resulting dose distributions were computed for all fractions. Different sets of criteria with varying dose thresholds for re-planning were investigated. All sets of criteria ensure equivalent target coverage of both CTVs, but vary in the tolerance threshold of the spinal cord.

**Results:**

The variations of the D95 and D2 in respect to the planned values ranged from -7% to +3% for both CTVs, and -2% to +6% for the spinal cord. Despite different correction vectors of the three IGRT strategies, the dosimetric differences were small. The number of fractions not requiring re-planning varied between 0% and 11% dependent on the applied IGRT correction strategy. In contrast, this number ranged between 32% and 70% dependent on the dosimetric thresholds, even though these thresholds were only gently modified.

**Conclusions:**

The more precise the planned dose needs to be maintained over the treatment course, the more frequently re-planning is required. The influence of different IGRT correction strategies, even though geometrically notable, was found to be of only limited relevance for the re-planning frequency. In contrast, the definition and modification of thresholds for re-planning have a major impact on the re-planning frequency.

## Background

Today, fractionated intensity modulated radiation therapy (IMRT) treatments for head and neck cancer (HNC) patients are predominantly performed adopting an image-guidance strategy. Fast online setup corrections can be performed immediately after image acquisition prior to irradiation. However, the flexibility of the head and neck (H&N) region frequently leads to deformations of the anatomy of the patient [1–3], deteriorating the applied dose distribution in regard to the planned one [4, 5]. Dealing with these deformations is an on-going challenge; increasingly they are compensated by re-planning [6], since deformations cannot be corrected by simple treatment couch shifts. Up to now, in most treatment planning systems (TPS) an automated recalculation of the dose distribution based on the daily control images is not implemented. Thus, the physicians have to decide whether re-planning is necessary based on their experience to interpret geometrical misalignments of image fusions. Therefore, an objective standardized concept to define criteria for re-planning is difficult to identify.

Currently used re-planning approaches and the accompanied efforts can differ considerably. These approaches include purely image-based methods that compare the alignment of anatomical structures between control-CT and planning-CT, or are based on time dependent protocols, e.g. re-planning on a weekly or midway basis [7], or a combination of both criteria. Also approaches enabling a daily re-planning scheme were proposed [6]. The cost and time aspects of these approaches differ, and it is still unclear how much effort is appropriate in clinical routine. First studies indicate an improvement in quality of life for the use of re-planning in HNC patients on a weekly basis [8].

Daily image guidance has the potential to assess the individual deformations of the patient’s anatomy necessary for a daily re-planning scheme. In principle, this enables selecting those fractions and patients with large deformations that mostly benefit from re-planning [9]. However, currently proposed methods for selection of re-planning criteria are based on geometrical measures only [9–11] although the impact of geometrical misalignments on the dose distribution is expected to be of greater importance.

The impact of the image-guided radiation therapy (IGRT) correction strategy preceding the decision whether re-planning is required is unclear. The choice of the method to derive the couch correction influences the resulting dose distribution [12] and the extent of its deterioration in regard to the planning phase. Applying a more optimized IGRT correction can be expected to re-position a patient more frequently within a tolerated uncertainty compared to less sophisticated IGRT methods, decreasing the overall time effort required by frequent re-planning. However, the extent of the dosimetric variations caused by different geometrical corrections is still rarely investigated [12, 13].

In this study we investigate the impact of different re-planning criteria and three IGRT correction strategies on the frequency of the necessity to re-plan. The chosen sets of criteria ensure equally precise target coverage but use different criteria for the spinal cord (SC).

## Methods

### Patients, target volumes, dose prescriptions

The CT data sets of six consecutively treated HNC patients were analysed. Written informed consent to include their anonymized data in retrospective studies was obtained from all patients. All patients were treated with a linear accelerator (Artiste, Siemens OCS, Erlangen, Germany) combined with an in-room, single slice spiral CT-scanner. All patients received daily kilo-voltage (kV) control-CTs. A total of 185 (28-32 per patient) CT-scans were evaluated. All patients were immobilized with a head mask and a vacuum pillow [3]. Four patients were treated postoperatively for oropharyngeal cancer; the remaining two patients received definitive radiation therapy for locally advanced hypopharyngeal cancer.

Two CTVs were delineated: The therapeutic CTV (tCTV) included the pre-surgical gross tumor volume, respectively the larynx and adjacent pre-vertebral fascia in the larynx cancer patients. The prophylactic CTV (pCTV) included the supraclavicular and cervical lymph nodes in all patients. The tCTV was extended by a CTV-to-PTV margin of 3 mm to define the tPTV. The larger pCTV was expected to be more prone to deformations than the tCTV. Thus, the pCTV was extended by a 5 mm CTV-to-PTV margin to define the pPTV.

In the IMRT planning process the maximum dose value to the spinal cord was limited to 45 Gy. A simultaneous integrated boost concept was used. The dose prescription to the tPTV was 70.4 Gy in 32 fractions (2.2 Gy/fraction) and 57.6 Gy to the pPTV. The dose calculation was performed with a superposition method [14].

### Simulation of the treatment course

#### a) Re-contouring of the VOIs

An automatic deformable image registration (DIR) algorithm based on a template matching technique was used to re-contour initially defined volumes of interest (VOIs) on each daily control-CT. The DIR method has been developed in-house and has demonstrated suitable accuracy for re-contouring in H&N anatomy [15]. All automatically propagated contours were reviewed and in case of obvious misregistrations (n = 10 out of 185 fractions) excluded from this analysis.

#### b) IGRT correction strategies

After the deformable re-contouring of the VOIs, the patient model still can show systematic positioning error. Therefore a treatment couch shift needs to be calculated from image scans to correct for this positioning error. Three IGRT correction strategies were simulated. All three strategies were modified to optimize translational degrees of freedom only, omitting the rotational components during the optimization process. This allowed the simulation of a target point correction (TPC), equivalent to a treatment couch shift.

The first strategy to derive a TPC uses rigid image registration (RIR), with a large registration box focused to the pCTV (RIR_box_large). The registration box limits the image region which is used in the registration process to focus the alignment to chosen structures in the patient’s anatomy. The RIR method is based on mutual information as similarity measure, and is used in the TPS clinically in our institution [16]. The second strategy uses the same rigid registration method with a small registration box enclosing the tCTV (RIR_box_small). The third method derives a correction from a displacement vector field (DVF) calculated by a DIR method (DIR_fit) [11]. In short, this method fits a rigid-body transformation to all vectors of the DVF within selected VOIs. Its objective function benchmarks the quality of the alignment (in terms of volume fractions) of the re-contoured VOIs to their shape and position in the planning-CT. All three strategies result in different translation vectors that are used to correct the target point of the initial plan file prior to dose re-calculation. The impact of a varying registration box on the alignment quality was investigated previously [9, 12].

#### c) Dose re-calculation on control-CTs

The dose re-calculation was performed on each control-CT after the adaptation of the target point according to the correction vector resulting from the three correction strategies. The same dose calculation method as in the planning process was used. The dose distributions were recalculated with constant monitor units determined in the planning phase. The dose volume histograms (DVHs) for the VOIs in each fraction were evaluated using the re-contoured VOIs and the re-calculated dose distributions. Care was taken that the DVH of the spinal cord was calculated considering the same cc-extent of the re-contoured VOI in all control-CTs.

#### d) Re-planning criteria with different dose thresholds

To evaluate the frequency of the necessity to re-plan four sets of re-planning criteria were investigated. These criteria define whether the dose distribution of the control-CT after TPC is acceptable or if re-planning is necessary. One set of criteria holds the dose value criteria (consisting of accepted dose intervals) for the both target volumes and the spinal cord volume. All sets contain three criteria: first two criteria monitor tCTV and pCTV coverage, allowing for a deviation of the D_95_ within an interval of -1% to +5% in respect to the planned value. The third criterion differs in the definition for the D_2_ of the SC, with D_x_ being the dose value at x% of the volume in the DVH of the corresponding organ. In the first set of criteria a fixed threshold is assigned for the SC in all patients. It is defined at 45 Gy regardless of its value in the planning phase. This means if the D_2_ exceeds 45 Gy, while the tCTV and pCTV coverage is within the defined interval, re-planning is required. If the D_2_ value is <45 Gy, independent of the individually planned D_2_, the TPC is accepted. In the second set of criteria the threshold is defined as the individual D_2_ value of the planning phase plus 2%, corresponding to about +1 Gy deviation. Again all D_2_ values below this threshold are accepted. In the third set of criteria an upper and a lower threshold around the planning D_2_ value is introduced. The accepted interval is set to ±2% in respect to the planned value, which roughly corresponds to ±1 Gy. The fourth set of criteria reduces the interval around the planned value to ±1% (about ±0.5 Gy), for a stricter dose maintenance.

## Results

**Figure 1:**
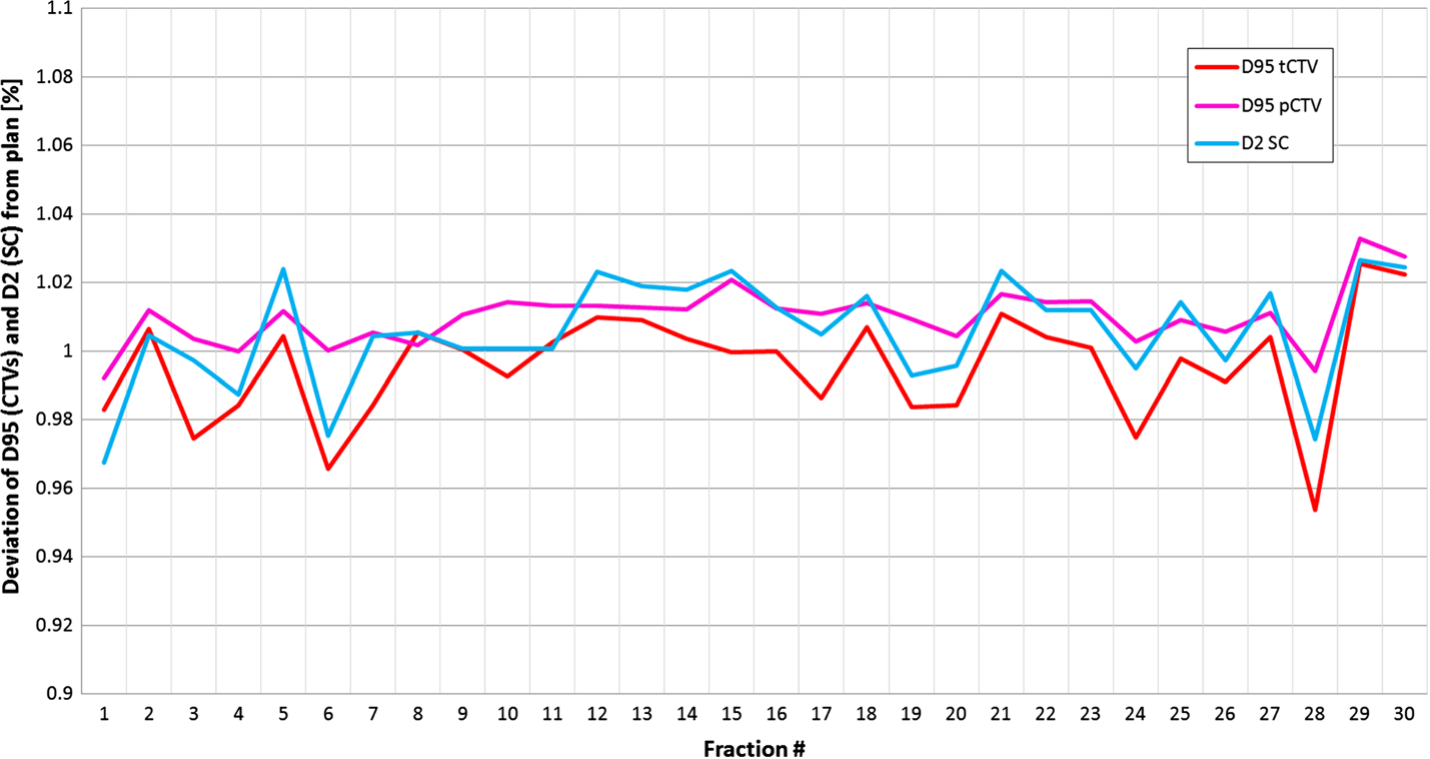
**Variation of DVH parameters over treatment course: DVH parameters are shown for both CTVs (pCTV: magenta, tCTV: red) and the spinal cord (blue) in patient #6 for the IGRT strategy RIR_box_large over the treatment course.** All values are normalized to the corresponding planned values.

Several DVH parameters are currently used in clinical routine to describe the quality of the treatment plan. The progress of the DVH parameters for both CTVs (D_95_) and the spinal cord (D_2_) for an exemplary patient (#6) and an exemplary IGRT strategy (RIR_box_large) is shown in Figure 1. The variation of the parameters in respect to the planned ones ranges between -5% and +3%. The variations for all patients and this correction strategy are summarized alongside the planned values in Tables 1 and 2.

**Table 1:** **Distributions of DVH parameters (target volumes): Overview of the DVH parameters over the treatment course for all patients (in case no re-planning is performed)**

Patient #	D95 [Gy] tPTV	D95 [Gy] tCTV			D95 [Gy] tCTV		D95 [Gy] pCTV	
	Planned	Min	Mean ± SD	Max	Planned	Min	Mean ± SD	Max
1	68.4	68.4	69.2 ± 0.4	69.8	55.2	55.3	56.2 ± 0.3	56.7
2	68.4	65.8	68.4 ± 0.7	69.1	53.6	51.9	52.9 ± 0.5	53.6
3	68.3	67.5	68.5 ± 0.5	69.3	53.6	53.5	53.9 ± 0.2	54.3
4	68.4	67.0	68.9 ± 0.7	70.3	54.4	53.5	54.6 ± 0.4	55.5
5	66.6	61.7	66.8 ± 1.4	68.1	54.7	54.6	55.0 ± 0.2	55.4
6	67.8	64.6	67.3 ± 1.0	68.5	54.3	53.9	54.8 ± 0.4	55.4

Abbreviations: *SC* spinal cord, *tCTV* therapeutic CTV, *pCTV* prophylactic CTV, *SD* standard deviation.

The values result from the RIR_box_large IGRT correction.

**Table 2:** Distributions of DVH parameters (spinal cord)*

Patient #	D2 [Gy] Spinal cord			
	Planned	Min	Mean ± SD	Max
1	44.6	44.1	44.8 ± 0.4	45.6
2	41.0	41.0	41.6 ± 0.3	42.5
3	38.7	37.8	38.7 ± 0.6	40.6
4	40.6	40.3	41.4 ± 0.6	42.7
5	42.9	41.9	42.4 ± 0.3	42.9
6	44.1	42.6	44.3 ± 0.7	45.1

*Same descriptions as in Table 1.

The influence of the IGRT strategy on the TPC vector is indicated by the distribution of the distances between the resulting vectors in each fraction of the different strategies. The range and the quartiles of the difference of the three IGRT strategies in between each other are summarized in Table 3. All three strategies use target point shifts only. The difference between these shifts varies considerably, with a difference in length of >2 mm in 50% of cases between each of two strategies. The correction vectors show a significant difference between the three strategies indicated by the p-values in a Friedman test of 0.003, 0.007, and <0.001 for x, y, and z component respectively.

**Table 3:** **Differences of correction vectors: Distributions of the distances between the target points if different IGRT correction strategies are applied (each fraction of all patients contributes equally)**

	Min	25% Quartile	Median	75% Quartile	Max
RIR_box_small vs. RIR_box_large [mm]	0.0	1.4	2.2	3.0	7.0
RIR_box_large vs. DIR_fit [mm]	0.0	1.3	2.2	3.0	5.9
DIR_fit vs. RIR_box_small [mm]	0.0	1.5	2.5	3.7	7.9

The dosimetric impact of the different TPC vectors on the DVH parameters of the considered VOIs was small. All three strategies resulted in comparable distributions of D_x_ values summarized in Table 4. Statistical analysis could not show a significant difference of the dose indicators for the spinal cord (p = 0.29). For the dose indicators for target volumes significant difference was found in at least one of the correction strategies (p ≤ 0.01), however, the amount of the deviations (<1%) is assumed not to be of major clinical importance. A set of criteria based on DVH parameters for VOIs allows defining to what extent dose deviations compared to the planned values are acceptable. Choosing such a set of criteria the frequency of the necessity to re-plan can be determined.

**Table 4:** **Differences of DVH parameters: Comparison of the distributions of DVH parameter resulting from three different IGRT correction strategies (each fraction of all patients contributes equally)**

	RIR_box_large: Δ _to plan_[%]	RIR_box_small: Δ[%]	DIR_fit: Δ[%]	p-value
	[−max, 25%, 50%, 75% + max]	[−max, 25%, 50%, 75%, + max]	[−max, 25%, 50%, 75%, + max]	
SC D2	[−3.2, −0.6, 0.5, 1.7, 5.4]	[−4.6, −0.5, 0.7, 1.7, 5.8]	[−4.1, −0.7, 0.3, 1.5, 4.1]	0.29
tCTV D95	[−7.3, 0.0, 0.5, 1.1, 2.7]	[−7.1, −0.2, 0.5, 1.1, 2.9]	[−7.3, −0.3, 0.3, 0.9, 2.1]	<0.01
pCTV D95	[−3.3, 0.0, 0.6, 1.2, 2.7]	[−3.5, −0.6, 0.3, 1.0, 3.0]	[−3.6, −0.4, 0.6, 1.3, 2.9]	0.01

Abbreviations: SC = spinal cord; tCTV = therapeutic CTV; pCTV = prophylactic CTV; RIR = rigid image registration; DIR = deformable image registration; Δ = deviation from the planned corresponding value.

Distribution scheme: [max negative deviation; lower quartile^(25%)^; median^(50%)^; upper quartile^(75%)^; max positive deviation].

**Figure 2:**
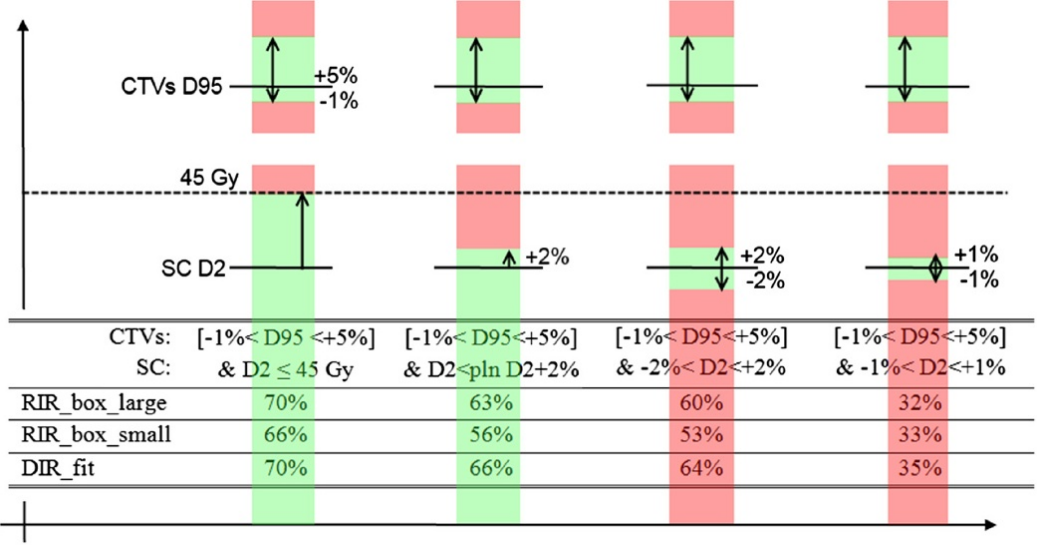
**Pass percentage for criteria sets: Number of fractions (in%) that have passed the four different re-planning criteria sets without the need of re-planning, dependent on the IGRT strategy.** The upper bars illustrate the thresholds for the CTVs. Black line: planned D95 value, green areas: tolerated deviation within the defined interval, red areas: unaccepted dose deviations. The lower bars represent the D2 thresholds that define the tolerance interval of the spinal cord.

**Figure 3:**
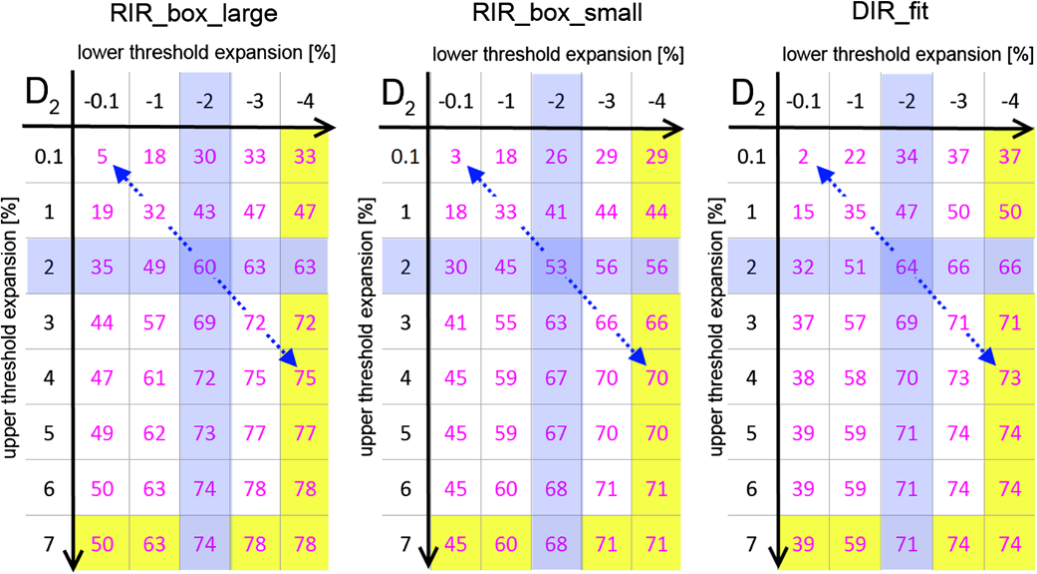
**Variation of pass percentage: Number of fractions (in%) that passed the re-planning criteria set depending on the varying interval definition and IGRT strategy.** The ±2% interval is marked in blue. Following the blue diagonal arrow corresponds to the symmetric expansion of the tolerance interval around the planned D2 of the spinal cord. The grey underlay indicates the interval size where the maximal number of fractions passing the interval is reached.

The frequency for adaptive re-planning depended to a greater extend on the re-planning criteria (e.g. 70% vs. 32% see Figure 2 (RIR_box_large)) than on the derivation of the daily IGRT correction vector (e.g. 53% vs. 64% (RIR_box_small vs. DIR_fit)). The least demanding set of criteria (D_2_ < 45 Gy) resulted in 30-34% of all fractions requiring a re-planning (mainly due to target coverage requirement not to spinal cord sparing). This means that at least two thirds of all fractions can sufficiently be corrected by either of the IGRT strategies. Maintenance of the D_2_ value below the individually planned one plus an upper tolerance of 2% decreases the number of fractions passing this second set of criteria without re-planning, and increases the frequency of re-planning to around 34-44%. For both criteria sets it has to be kept in mind, that even though the thresholds in principle allow a decrease of the D_2_ value down to 0 Gy, this is not probable to happen. In fact, the D_2_ values do not fall below -3% (~-1.5 Gy), meaning that a interval of -3% < D_2_ < +2% results in the same re-planning frequency as the second set of criteria using an interval of -100% < D_2_ < +2%. This is shown in Figure 3, where the probability for a fraction to pass the set of criteria dependent on the size of the interval converges to a stable level (indicated by the underlying yellow layer). Further increase of the tolerance interval for the spinal cord beyond the marked yellow layer (e.g. <54 Gy) does not result in all fractions passing, since the criteria for the target volumes is unchanged. These criteria still identify 22-29% of the fractions with unacceptable target coverage after the applied IGRT repositioning strategy. Narrowing the tolerance interval defined by the upper and lower threshold for the D_2_, the re-planning frequency increases up to 68% for the ±1% interval.

A more general overview of the frequency of the re-planning necessity and its sensitivity to gentle variations of the SC D_2_ criterion, which defines an interval of acceptable deviations from the planning D_2_ value, are plotted in Figure 3. This figure shows the percentage of fractions that passed depending on the applied upper and lower threshold in respect to the planning D_2_ value.

## Discussion

In this study, the dosimetric consequences applying three different IGRT correction strategies are investigated in deforming H&N anatomy with regard to the ability to maintain the prescribed dose over a treatment course without re-planning. Additionally, the effect of different thresholds on the frequency to re-plan is evaluated.

It is known that the size and site of the region used for the image registration process influences the target point correction vector and the alignment of different structures [12]. Likewise correction vectors, computed by different methods, result in a different alignment quality of the VOIs after re-positioning [11] and impact the resulting dose distribution in the patient. To analyze these dosimetric effects is of interest, because different TPS vendors use different implementations of registration algorithms to derive these vectors, which again can show big variations even if same input data are used [17]. The three IGRT strategies evaluated in this study differ in the region of registration or computational method used for image alignment. They are exemplary chosen to represent varying resulting target point corrections. It needs to be kept in mind that the chosen correction strategies do not cover the whole range of possible variations in clinically used correction strategies. However, they are used to demonstrate the impact of the shift variations and do result in geometrically different correction vectors comparable to the variability found in other studies comparing correction vectors of different commercially available treatment planning systems [17]. In the treatment planning process we considered it particularly important to achieve a high PTV coverage of both target volumes. Despite the differences in the re-positioning vectors, the observed variations of the DVH parameters (D_95_, D_02_) between the fractions were small, with a standard deviation of less than 1.4 Gy for all analyzed VOIs (Tables 1 and 2). Yet, the deformations of these patients were shown to be comparable to those of other HNC collectives [3]. A previous study has reported dosimetric variations dependent on different repositioning parameters that have been derived by a manual alignment of different substructures in the H&N region [13]. Compared to our results, slightly higher relative variations have been observed, which can be due to focusing on smaller and more distant substructures for alignment and considering also rotational components in the latter study.

While small dosimetric differences resulting from the three IGRT strategies only had a small influence on the necessity to re-plan, the chosen re-planning criteria set had a big impact. The selection of the thresholds reflects clinical considerations: To ensure optimal target volume coverage over the treatment course, the acceptable dose deviation for re-planning was set to be a narrow interval [-1 to +5%] around the planned D_95_ value. This criterion is used for both target volumes in all four re-planning criteria sets. The sets of criteria differ only in the criterion for the D_2_ of the spinal cord (Figure 3). The first set of criteria assures that the D_2_ of the spinal cord should not exceed 45 Gy independent of the individually planned value for this organ at risk. The second set of criteria, which uses the individual D_2_ value as threshold, is of interest in patients requiring re-irradiation. Due to their previous dose exposure it can be important to strictly maintain the planned D_2_ value in these patients with certain accuracy. The third and fourth sets of criteria are even stricter and demand dose maintenance within an interval, not allowing the D_2_ value to de- or increase excessively since a significant decrease might be associated with a dose increase in the surrounding tissue.

Applying these sets of criteria in our patient cohort, it was observed that already small modifications of the criteria for D_2_ of the spinal cord caused considerable variations in the number of fractions that required re-planning (Figure 2).

The availability of routine use of volumetric imaging not only allows to perform IGRT correction strategies, but also revealed an increasing demand for adaptive re-planning in fractionated courses [18]. Our results confirm the expectation that the re-planning frequency needs to be increased, the more precise the planned dose value for the spinal cord needs to be maintained. The impact of the IGRT strategies on the dosimetric outcome was found of only limited relevance for clinical practice if a deviation of the D_2_ value up to 1 Gy is likely to be tolerated. Similar dependences were found for the D_max_ (SC) or D_98_ (target volumes) values (data not shown). Only if a precise maintenance of the planned dose is essential within a 2% interval (<1 Gy deviation), the influence of the different IGRT strategies, which result in slightly different alignments of the substructures, becomes visible. In this case the DIR_fit strategy, derived from more image information than the competing strategies, showed to be slightly superior with a pass rate of 64% (Figure 2). The RIR_box_small strategy, where the rigid registration is focused to the smaller high-dose-region showed to be inferior with a pass rate of 53%. The difference between the DIR_fit and RIR_box_large strategy is not very pronounced. It might become more important when also rotational components of the correction parameters are taken into account for re-positioning.

## Conclusions

Despite of daily applied patient setup corrections based on image-guidance, a fraction of HNC patients requires a plan adaptation in selected fractions, due to the deformability of this anatomical region. The more precise the planned dose needs to be maintained over the treatment course, the more frequently re-planning is required. The influence of different IGRT correction strategies, even though geometrically notable, was found to be of only limited relevance for the re-planning frequency. Only if maintenance of the planned values of less than 1 Gy is essential, careful selection of the IGRT strategy became relevant in our patients. In contrast, the definition and modification of thresholds for re-planning have a major impact on the re-planning frequency. These results implicate that more work on the investigation and standardization of the re-planning criteria needs to be done.

### Consent

Written informed consent was obtained from all patients for the publication of this report and any accompanying images.
